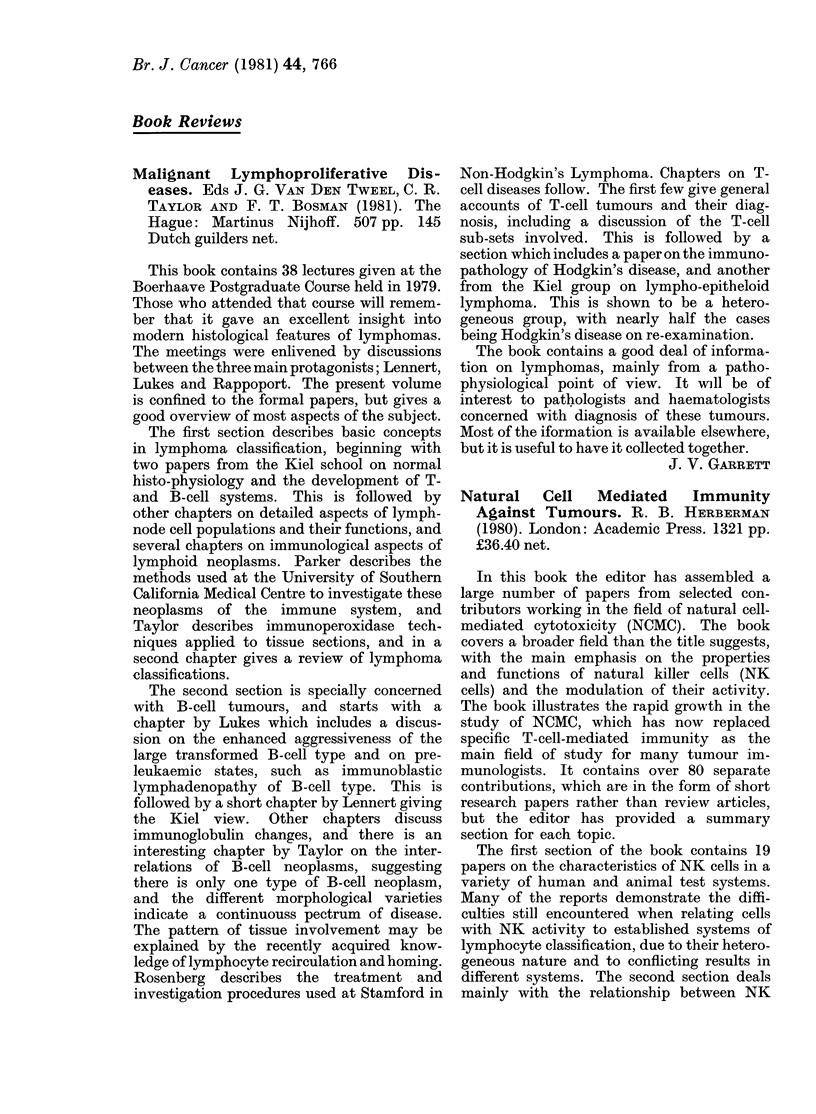# Malignant Lymphoproliferative Diseases

**Published:** 1981-11

**Authors:** J. V. Garrett


*Br. J. Cancer* (1981) **44**, 766

### *Book Reviews*

**Malignant Lymphoproliferative Diseases.** Eds J. G. VAN DEN TWEEL, C. R. TAYLOR AND F. T. BOSMAN (1981). The Hague: Martinus Nijhoff. 507 pp. 145 Dutch guilders net.

This book contains 38 lectures given at the Boerhaave Postgraduate Course held in 1979. Those who attended that course will remember that it gave an excellent insight into modern histological features of lymphomas. The meetings were enlivened by discussions between the three main protagonists; Lennert, Lukes and Rappoport. The present volume is confined to the formal papers, but gives a good overview of most aspects of the subject.

The first section describes basic concepts in lymphoma classification, beginning with two papers from the Kiel school on normal histo-physiology and the development of T- and B-cell systems. This is followed by other chapters on detailed aspects of lymph-node cell populations and their functions, and several chapters on immunological aspects of lymphoid neoplasms. Parker describes the methods used at the University of Southern California Medical Centre to investigate these neoplasms of the immune system, and Taylor describes immunoperoxidase techniques applied to tissue sections, and in a second chapter gives a review of lymphoma classifications.

The second section is specially concerned with B-cell tumours, and starts with a chapter by Lukes which includes a discussion on the enhanced aggressiveness of the large transformed B-cell type and on pre-leukaemic states, such as immunoblastic lymphadenopathy of B-cell type. This is followed by a short chapter by Lennert giving the Kiel view. Other chapters discuss immunoglobulin changes, and there is an interesting chapter by Taylor on the inter-relations of B-cell neoplasms, suggesting there is only one type of B-cell neoplasm, and the different morphological varieties indicate a continuouss pectrum of disease. The pattern of tissue involvement may be explained by the recently acquired knowledge of lymphocyte recirculation and homing. Rosenberg describes the treatment and investigation procedures used at Stamford in Non-Hodgkin's Lymphoma. Chapters on T-cell diseases follow. The first few give general accounts of T-cell tumours and their diagnosis, including a discussion of the T-cell sub-sets involved. This is followed by a section which includes a paper on the immunopathology of Hodgkin's disease, and another from the Kiel group on lympho-epitheloid lymphoma. This is shown to be a heterogeneous group, with nearly half the cases being Hodgkin's disease on re-examination.

The book contains a good deal of information on lymphomas, mainly from a pathophysiological point of view. It wıll be of interest to pathologists and haematologists concerned with diagnosis of these tumours. Most of the iformation is available elsewhere, but it is useful to have it collected together.

J. V. GARRETT